# Editorial: Computational methods and systems to support decision making in pharmacovigilance

**DOI:** 10.3389/fdsfr.2023.1188715

**Published:** 2023-04-21

**Authors:** Taxiarchis Botsis, Robert Ball, G. Niklas Norén

**Affiliations:** ^1^ The Sidney Kimmel Comprehensive Cancer Center, Johns Hopkins University School of Medicine, Baltimore, MD, United States; ^2^ United States Food and Drug Administration, Silver Spring, MD, United States; ^3^ Uppsala Monitoring Centre, Uppsala, Sweden

**Keywords:** pharmacovigilance, decision support, artificial intelligence, quality assurance, real-world data

The World Health Organization defines pharmacovigilance (PV) as “the science and activities related to the detection, assessment, understanding, and prevention of adverse events or any drug-related problem.” (World Health Organization, 2023) Government agencies, clinical institutions, the pharmaceutical industry, and other entities manage PV programs as vital safeguards for supporting the early detection and analysis of safety signals. These rely primarily on expert judgment and concrete business practices specific to each organization (Ball and Dal Pan, 2022). Computational methods and decision-support systems may enable more timely, consistent, and comprehensive analysis and processing of real-world data (RWD) and may free up time for domain experts to focus on higher-value contributions. As a positive side-effect, their development can help standardize business practices by specifying the steps human reviewers follow. In this Research Topic issue, we primarily invited papers that assess contributions to PV which may improve efficiency in established business practices, minimize manual effort, and maximize the quality of human decision-making by enhancing existing processes transparently. We also welcomed case studies of method implementation and perspectives that might significantly contribute to the domain.

Primary use cases in PV, such as case processing and prioritization or signal detection and evaluation, incorporate several steps that the complete decision-support system should augment. As data, user-related, and other challenges of the entire workflow vary, most efforts, to this date, have delivered solutions addressing more narrowly defined tasks. Historically, computational methods were initially proposed and deployed to enable signal detection and analysis in large databases using only structured data. More recently, several studies have presented methods for processing unstructured free texts, prioritizing case reports for clinical and regulatory review, and improving data quality in spontaneous reporting systems. For example, Painter et al. conducted a meta-analysis of a recent scoping review on machine learning in PV (Kompa et al., 2022) and found that the pharmaceutical industry mainly applied machine learning to process RWD and social media. They also highlighted the need to develop consistent systems that can learn and incorporate human-in-the-loop mechanisms and called for best practices for adopting and validating these systems.

To build effective solutions, existing workflows and business practices must be understood. These are rarely documented in stepwise algorithmic forms that can easily be translated into programmatic tools. It is, therefore, necessary to take a step back and, in close collaboration with the end users, identify the overall desired framework for a selected workflow. The papers in this Research Topic highlight several important aspects of this. Fong et al. pursued a strategy to support the medication error categorization workflow in the US Food and Drug Administration Adverse Event Reporting System (FAERS). Interestingly, they first defined a framework that helped them clarify the use case and then developed a prototype user-centered Artificial Intelligence (AI) application based on this framework. This process might work both ways, i.e., the first version of the framework implemented in a prototype tool could be informed by the pilot use of this prototype and, subsequently, updated to support new rounds of development.

Kreimeyer et al. focused on detecting duplicate cases in FAERS using the complete structured and free-text information from FAERS Individual Case Safety Reports (ICSRs). They built the confidence required to operationalize it by engaging safety reviewers in developing, evaluating, and validating their deduplication as a supplement to human review. Dang et al. developed a natural language processing approach to extract four demographic variables (gender, weight, ethnicity, and race) from FAERS ICSR narratives and complete the missing values in the corresponding structured fields. While their approach excellently retrieved ethnicity, gender, and race, it would meaningfully improve only gender information in the actual practice, as reporters infrequently provide weight, ethnicity, or race in the FAERS narratives. This demonstrates that operationalizing an AI solution must evaluate multiple factors, not solely performance.

Dimitriadis et al. presented an open-source platform that integrated ICSRs, synthetic clinical data, social media data from Twitter, and scientific literature from PubMed. Their prototype web-based tool allowed for various analyses in an interactive user interface. However, a comparison with the EudraVigilance Data Warehouse and Analysis System (EVDAS) provided by the European Medicines Agency revealed that PV experts preferred the existing technology. This study illustrates how implementing new tools in an actual work environment requires significant effort in presenting complete and validated systems and educating end users on their application.

When generative AI hits the headlines and heated discussions ensue (Stokel-Walker, 2023), it is essential to emphasize that PV is a tightly controlled, multi-step process. The rapid improvement of large language models across multiple applications should benefit PV, but to build trust with PV experts, we must systematically engage with users when collecting requirements and designing decision-support systems. Incorporating AI and other sophisticated algorithms into these systems requires knowledgeable interdisciplinary teams, including end-users and software developers, and several validation rounds aligned with the supported tasks and business workflows. Some of the studies in our Research Topic followed this strategy. This is the recipe for success in most cases as long as the right expectations are set from the beginning and the proposed solutions do not bring dramatic changes to existing workflows without proven benefit. Including a human-in-the-loop further ensures that a system’s limitations are carefully examined and that a quality assurance process to control them is in place. Ultimately, as no automated solution is likely to be perfect, decision-support systems must be constructed to take advantage of the respective strengths of machines and humans to improve efficiency, improve quality, and add value beyond what each might achieve on their own.
